# Indoor- and Outdoor-Generated Particles and Children with Asthma

**DOI:** 10.1289/ehp.113-a581a

**Published:** 2005-09

**Authors:** Hanns Moshammer

**Affiliations:** Institute of Environmental Health Medical University of Vienna, Vienna, Austria, E-mail: hanns.moshammer@meduniwien.ac.at

In their article “Pulmonary Effects of Indoor- and Outdoor-Generated Particles in Children with Asthma,” Koenig et al. (2005) made use of their really interesting model that enables them to discern exposure from indoor- and outdoor-generated particles. They concluded that

The ambient-generated component of PM_2.5_ [particulate matter ≤2.5 μm in aerodynamic diameter] exposure is consistently associated with increases in eNO [exhaled nitric oxide] and the indoor-generated component is less strongly associated with eNO.

This finding should not lead to the assumption that particles generated indoors are in general not able to induce endogenous NO production. The authors themselves pointed out one limitation of their study:

Children in the Seattle panel study spent an average of 66% of their time indoors at home and 21% indoors away from home (primarily at school) … (Koenig et al. 2005)

However, contribution of indoor sources to PM exposure was only estimated on the basis of measurement data from the subjects’ residences. This could have led to uncertainties in the exposure assessment, biasing the effect estimates toward null.

I also want to mention the great variability in possible indoor sources of PM. In their article, Koenig et al. (2005) provided no information on the smoking status of household members. If the 19 children under study lived in nonsmoking households, the results might be true for this setting but not for others.

Finally, I suggest that time of measurement and exposure should be considered. If the children attend school in the morning, they might go home (maybe in high traffic) at noon or early afternoon for lunch.

[NO] samples were collected in the afternoon or early evening at the child’s residence. Children were asked to forgo food intake for 1 hr before collection of exhaled breath.

If NO production peaks several hours after exposure, it could be possible that the children’s exposure on their way home from school was the most influential one (not because of the origin of the particles but because of the study’s lag structure). It would be interesting and rather rewarding to study the short-term lag structure between PM exposure and both exhaled NO and lung function [for which Koenig et al. (2005) found an association with exposure due to sources in the residents’ homes]. I would expect an increase of exhaled NO to be a rather late reaction to inflammatory stimuli. For example Rolla et al. (2004) reported that after aspirin inhalation by subjects with aspirin-inducible asthma, NO increased significantly reaching the peak value 4 hr after bronchoconstriction.
